# Synergistic DNA-adenovirus prime-boost immunization eliminates metastatic colorectal cancer by inducing high avidity effector CD8+ T cells

**DOI:** 10.1186/2051-1426-2-S3-P66

**Published:** 2014-11-06

**Authors:** Bo Xiang, Peng Li, Micheal S Magee, Scott A Waldman, Vitali Y Alexeev, Adam E Snook

**Affiliations:** 1 Departments of Pharmacology & Experimental Therapeutics, Thomas Jefferson University, Philadelphia, PA 19107, USA; 2 Department of Anatomic Pathology, Huntsman Cancer Hospital, Salt Lake City, UT 84112, USA; 3 Dermatology & Cutaneous Biology, Thomas Jefferson University, Philadelphia, PA 19107, USA

## 

Heterologous prime-boost immunizations with plasmid DNA and viral vaccines synergize to produce effective immune responses against tumor and infectious disease antigens. Yet, mechanisms underlying their synergy remain incompletely defined. Here, we used the colorectal cancer (CRC) antigen guanylyl cyclase C (GUCY2C) to explore the synergy of DNA-Ad5 (adenovirus type 5) prime-boost immunizations. GUCY2C is selectively expressed by intestinal epithelial cells and its expression is maintained by metastatic CRC cells. The compartmentalized expression of GUCY2C, normally in the mucosa but extending systemically in metastases, makes GUCY2C a target for CRC immunotherapy. Adenoviral vector expressing GUCY2C (Ad5-GUCY2C) is safe and effective in mice, producing immune responses and antitumor immunity, but heterologous prime-boost strategies may be required to maximize therapeutic efficacy. DNA vaccines are advantageous in heterologous prime-boost strategies reflecting the absence of vector-specific immunity which limits repetitive viral delivery. However, low transfection rates and immunogenicity characterize DNA vaccine strategies. Here, combining CCL20 and CCL21 as chemokine adjuvants to recruit dendritic and T cells, respectively, with electroporation (EP) of GUCY2C-expressing plasmids to increase transfection rates, optimizes GUCY2C immune responses. Primary immunization with DNA plasmids encoding chemokines and GUCY2C followed by boosting with Ad5-GUCY2C (DNA-Ad5) produced curative antitumor responses, in contrast to immunization with either component alone. Interestingly, DNA-Ad5 produced only a modest increase (<2-fold) in the magnitude of GUCY2C-specific CD8^+ ^T cell responses without changes in polyfunctional T cell responses. However, DNA-Ad5 dramatically improved CD8^+ ^T cell sensitivity by increasing T cell receptor (TCR) functional avidity for GUCY2C (~20-fold). Preventing enhanced CD8^+ ^T cell TCR avidity, while preserving the enhanced CD8^+ ^T cell quantity, eliminated antitumor efficacy. Thus, a GUCY2C DNA-Ad5 prime-boost strategy synergizes to produce highly potent CD8^+ ^T cell responses and therapeutic CRC activity, which may translate to patients at risk for metastatic CRC.

